# Introducing FMEA plus method for comprehensive safety risk assessment in the steel industry

**DOI:** 10.1371/journal.pone.0331748

**Published:** 2025-10-09

**Authors:** Ahmad Soltanzadeh, Esmaeil Zarei, Mohsen Mahdinia, Kiana Hosseinzadeh, Mohsen Sadeghi-Yarandi

**Affiliations:** 1 Research Center for Environmental Pollutants, Department of Occupational Safety & Health Engineering, Faculty of Health, Qom University of Medical Sciences and Health Services, Qom, Iran; 2 Department of Safety Science, College of Aviation, Embry-Riddle Aeronautical University, Prescott, Arizona, United States of America; 3 Department of Occupational health and safety engineering, School of Public Health, Hamadan University of Medical Sciences, Hamadan, Iran; 4 Health in Emergency and Disaster Research Center, Social Health Research Institute, University of Social Welfare and Rehabilitation Sciences, Tehran, Iran; Istanbul University: Istanbul Universitesi, TÜRKIYE

## Abstract

Addressing the challenge of employing a comprehensive risk analysis approach that effectively captures and quantifies all contributing factors remains a significant endeavor in both academic research and practical field applications. This study endeavors to fill this gap by introducing a practical safety risk assessment approach, named the FMEA+ method, grounded in the conventional Failure Modes and Effects Analysis. To construct a comprehensive taxonomy that encompasses the contributing factors within each dimension of risk, a three-stage Delphi study engaged 35 Subject Matter Experts (SMEs). The Fuzzy Analytical Hierarchy Process (FAHP) was employed to acquire knowledge and assign weights to the factors and sub-factors. The validation and reliability assessment of the developed taxonomy included evaluating the Content Validity Ratio (CVR), Content Validity Index (CVI), and Cronbach’s alpha coefficient, yielding values of 0.77, 0.91, and 0.86, respectively. Independent peer reviews and reality checks further substantiated the credibility of the proposed taxonomy. The introduced safety risk assessment algorithm, FMEA + , derived from the FMEA technique, comprises three main factors and 12 sub-factors. The final normalized weights for the three factors—occurrence, severity, and detectability—were determined to be 0.337, 0.348, and 0.315, respectively. In the three factors of occurrence, severity, and detection, the most important sub-factors identified were human reliability, human injury, and technical inspection, respectively. This proposed taxonomy serves as a foundational tool for facilitating informed decision-making and the effective implementation of risk mitigation strategies. The application of this innovative approach offers a scientific alternative to traditional FMEA methods within similar industries, addressing existing challenges in a more comprehensive and nuanced manner.

## 1. Introduction

In general, risk assessment is a crucial component of a cycle known as risk management, which encompasses the following steps: (1) hazard identification (HAZID), (2) risk assessment, (3) risk evaluation, and (4) implementation of control measures. Risk assessment is regarded as the core of an effective risk management system [1–6]. Improving workplace safety typically begins with a risk assessment that examines potential hazards. The first step in this process is risk analysis, which is followed by risk estimation. According to ISO 12100, risk analysis consists of three steps: identifying constraints, identifying hazards, and risk estimation [6,7].

Risk identification and assessment are essential components of any safety management system. Risk assessment is a logical approach to hazard assessment that identifies hazards and their potential consequences for people, materials, equipment, and the environment. Risk assessment methods enable industrial facilities to reduce risks, establish acceptable risk levels, implement inspection and maintenance policies, and establish standard operating procedures (SOP) [1,8–10].

Risk assessment can be performed in many ways. Currently, a variety of quantitative and qualitative risk assessment methods are used in a wide range of industries, so approximately, there are more than 70 types of risk assessment methods [11]. Risk assessment methods can be categorized into quantitative and qualitative approaches based on the parameters utilized. Quantitative methodology employs statistical or numerical data to evaluate the degree of risk. In contrast, qualitative methodology relies on observations, categorical ratings, and non-numerical values to assess risk levels. Most risk assessment tools incorporate two key parameters: Severity and Probability. Numerous risk assessment tools are available, including Safety Checklists, Job Safety Analysis (JSA), Hazard and Operability Study (HAZOP), Failure Mode and Effects Analysis (FMEA), and Fault Tree Analysis (FTA), among others. To effectively prevent accidents, it is essential to select an appropriate risk assessment method tailored to the specific conditions of industries and organizations, as well as the nature of work activities. Given the complexity of accidents across various sectors, many countries, organizations, and industrial holdings are developing and adapting risk assessment techniques to enhance their accuracy and compatibility with industrial processes and the diverse nature of job tasks [2,12–14].

Failure Mode and Effects Analysis (FMEA) is widely utilized across various industries to assess safety risks. This method was first introduced by the aerospace industry (NASA) in 1960. FMEA identifies, evaluates, and controls fault conditions, as well as the causes and effects of potential faults within a system. It assesses failure modes using the Risk Priority Number (RPN) index, which is calculated by multiplying three parameters: Probability of Occurrence, Severity of Impact, and Detectability. A higher RPN value indicates a greater risk associated with the failure mode. Bottom-up failure analysis identifies failure modes, analyzes their causes, and maps their effects at all levels, from components to systems to processes. However, one of the main limitations of this method lies in the nature of the parameters evaluated in the three components and their weighting. These factors can influence the determination of risk levels and the prioritization of corrective measures across different industries [11,15,16].

The present study aims to develop a semi-quantitative risk assessment technique based on the Failure Mode and Effects Analysis (FM&EA) method, conducted within the steel industry. The steel industry is a significant and high-risk sector globally. Employees in this industry are exposed to high risks due to the types of energy utilized, work processes, environmental conditions, and various hazardous factors present in the workplace [17]. There are numerous safety risks associated with the steel industry, including chemical, metallurgical, electrical, mechanical, construction, mining, geological, transportation, and logistical hazards. It is essential to recognize that the various dangers of working in this industry encompass exposure to dust and fumes, excessive noise, confined workspaces, heat stress, harmful gas leaks, steel arc processes, hot metal spills, explosions, and fires [18].

Implementing risk management and assessment tailored to the specific working environment is one of the most effective strategies for reducing occupational accidents. This approach involves identifying, assessing, and evaluating all potential risks, followed by the implementation of appropriate control measures based on the results of the risk assessment. The steel industry, in particular, experiences a wide variety of accidents, complicating the determination of the probability and severity of these events due to the nature of the risk assessment process and the types of accidents that occur. Consequently, utilizing an appropriate tool that addresses the identified risk levels will be highly beneficial.

### 1.2 Main research questions

Some potential research questions for this study included the following:

1. What are the limitations of current safety risk assessment methods used in steel production?
2. How can the Delphi method be used to refine and validate the FMEA-based risk assessment framework?
3. How can FAHP be employed to determine the relative weights of different criteria and sub-criteria within the FMEA-based risk assessment framework?
4. What are the most significant criteria and sub-criteria to consider when using FAHP to prioritize safety risks in steel production?
5. What sub-factors can be effective in estimating the occurrence, severity, and detection components?
6. What are the most important factors and sub-factors in risk estimation using the FMEA^+^ method in the steel industry?

Ultimately, due to the lack of a comprehensive tool capable of examining the critical components and parameters that affect the likelihood and severity of accidents across various industries—especially in the steel industry, which is one of the largest and most vulnerable sectors worldwide—this study aimed to develop a semi-quantitative safety risk assessment method based on the FMEA method, referred to as the FMEA+ method.

### 1.3 Literature review

Multi-Criteria Decision-Making (MCDM) methods, such as the Analytic Hierarchy Process (AHP), the Technique for Order of Preference by Similarity to Ideal Solution (TOPSIS), and the Preference Ranking Organization Method for Enrichment Evaluation (PROMETHEE), have been proposed to improve the decision-making process in FMEA. AHP, for example, enables decision-makers to organize subjective judgments and preferences by assessing the relative importance of various criteria. Its pairwise comparison technique aids in prioritizing failure modes based on multiple factors, including severity, occurrence probability, detectability. TOPSIS provides a methodology for ranking failure modes by evaluating their proximity to the ideal solution and their distance from the negative ideal solution, taking into account multiple criteria and their relative importance. This reduces subjectivity and bias in determining the Risk Priority Number (RPN). Secondly, MCDM methods allow for sensitivity analysis, enabling decision-makers to assess the impact of changes in criteria weights. This enhances the flexibility and adaptability of the decision-making process. Lastly, the application of MCDM methods in FMEA promotes transparency and accountability in risk assessment and management. The rationale behind the selection of risk mitigation measures becomes clearer, allowing stakeholders to better understand and evaluate the decision-making process [19–24].

So far, many researchers have tried to expand FMEA by employing various methods, including fuzzy logic systems, Dempster–Shafer theory, Multi-Criteria Decision Making (MCDM) methods, the Analytic Hierarchy Process (AHP), and the Technique for Order of Preference by Similarity to Ideal Solution (TOPSIS). These studies began in 1999. By 2018, 44 studies had been published in the field of FMEA expansion. The methods developed have been applied across diverse sectors, including manufacturing, mining, aerospace, healthcare, process, and electronics industries [25].

Several studies have addressed the limitations of traditional FMEA by incorporating fuzzy logic [26,27]. Fuzzy logic allows for the representation of uncertainty and vagueness in expert judgments, leading to more realistic risk assessments [28]. This is particularly relevant when dealing with subjective assessments of severity, occurrence, and detection, where precise numerical values may be difficult to assign. The integration of fuzzy logic into FMEA, often referred to as Fuzzy-FMEA, uses fuzzy numbers to represent the S, O, and D parameters, thereby capturing the inherent uncertainty in evaluations [29].

Further enhancements include combining Fuzzy-FMEA with other Multi-Criteria Decision Making (MCDM) methods, such as the Best-Worst Method (BWM) [26] or Technique for Order Preference by Similarity to Ideal Solution (TOPSIS) [30]. These methods provide more sophisticated ways to weight the FMEA parameters and prioritize risk factors, leading to more accurate and reliable risk assessments. The integration of Bayesian networks with Fuzzy-FMEA allows for consideration of dependencies between failure modes, providing a more comprehensive risk assessment [26]. In addition, stochastic approaches have been introduced to FMEA, incorporating random numbers to represent the uncertainty in expert evaluations, leading to more robust and reliable results. Furthermore, the use of hesitant fuzzy sets in FMEA allows for explicit handling of situations where experts are hesitant in their assessments, leading to a more nuanced and accurate risk assessment [27].

FAHP is an extension of the traditional Analytical Hierarchy Process (AHP) that incorporates fuzzy logic to handle uncertainty and imprecision in decision-making [31]. AHP is a hierarchical multi-criteria decision-making method that allows for the systematic comparison and weighting of different criteria. FAHP extends this by using fuzzy numbers to represent the pairwise comparisons between criteria, which allows for the incorporation of expert judgment and the handling of uncertainty [32].

FAHP is frequently integrated with FMEA and the Delphi method to develop comprehensive safety risk assessment models [31]. The Delphi method can be used to determine the criteria and sub-criteria for the FAHP model, while FAHP can then be used to weight these criteria and sub-criteria based on expert judgment. The resulting weights can then be used to calculate the overall risk associated with each failure mode in the FMEA, providing a more comprehensive and robust assessment of risk [33]. The use of FAHP helps overcome the subjectivity inherent in traditional FMEA, by providing a more structured and objective way to weight the different risk factors. Moreover, FAHP can address the shortcomings of traditional AHP by handling the inconsistency of judgment matrices and quantifying system risk effectively [32]. The combination of FAHP and other MCDM methods, like TOPSIS, further enhances the accuracy and reliability of the risk assessment process [34]. In some cases, a nonlinear FAHP model can be used to better capture the non-linear relationships between risk factors, leading to more accurate risk assessment [35].

Several studies demonstrate the successful application of integrated FMEA-Delphi-FAHP methodologies in various contexts. Teng Ji et al. [31] proposed a safety risk assessment method for large and complex bridges during construction, using the Delphi method to identify risk factors, FAHP to calculate weights, and factor analysis to select representative factors. Mohsen Sadeghi-Yarandi et al. [2] developed an Electrical Industry Safety Risk Index (EISRI) in the electricity power distribution industry based on FAHP, using a three-stage Delphi study to determine the main components and parameters. Ziwen Wang and Yuan Yuan [33] established a visualized digital construction safety risk model for high-pile wharves based on a combination of fuzzy comprehensive evaluation (FCE) and AHP, incorporating BIM model parameters. These studies highlight the versatility and effectiveness of the integrated approach in addressing complex safety risks across different domains.

Recent studies have underscored the importance of advanced fuzzy-hybrid approaches in safety risk assessment. For example, the novel intuitionistic fuzzy-hybrid-modified TOPSIS method provides improved uncertainty quantification and risk prioritization in complex systems [14]. Similarly, frameworks that integrate fuzzy logic with decision-making models have been developed to enhance risk analysis and mitigation. These frameworks focus on aggregating expert judgments and managing imprecise data [2,36,37]. Furthermore, hybrid approaches to uncertainty modeling in safety systems have demonstrated superior capabilities in integrating multiple sources of uncertainty and enhancing decision quality, reflecting resilience-oriented perspectives in safety assessment.

As demonstrated, despite the implementation of advanced computational methods, particularly in process industries, there is still a lack of a user-friendly structured approach. Many studies overlook sub-components in addition to the three primary components of this method, including occurrence, severity, and detection. Furthermore, there are no established equations to calculate the scores for each sub-component or the final risk priority number. Additionally, some researches risk factors as equally important, which may not accurately reflect their varying impacts. To address these limitations, the present study was carried out.

## 2. Method

This study aimed to develop a new semi-quantitative technique for safety risk assessment in industries, based on Failure Mode and Effects Analysis (FMEA) and utilizing the Delphi method, Analytical Hierarchy Process (AHP), and fuzzy logic in 2024–2025. The study began on December 10, 2024, and concluded on April 10, 2025. The study was conducted at a large-scale steel manufacturing plant in Qom Province, Iran, which is characterized by complex and hazardous operational processes. These processes include high-temperature metal processing, heavy machinery operation, and extensive manual interventions. Key steps in the manufacturing process encompass raw material handling, melting, casting, and finishing.

All methods were conducted in accordance with the relevant guidelines and regulations set forth by the Ethics Committee of Qom University of Medical Sciences. Prior to the study, informed consent was obtained from all participants. This study was approved by the Ethics Committee of Qom University of Medical Sciences (Ethics approval code: IR.MUQ.REC.1403.184). Written informed consent was obtained from all participants in the Delphi panel prior to their involvement. Participants were thoroughly informed about the study’s objectives, methods, confidentiality measures, assurances of anonymity, the voluntary nature of their participation, and their right to withdraw at any stage without facing any repercussions.

### 2.1 Failure mode and effects analysis (FM&EA)

This technique identifies and determines the impact of major failure modes on the reliability, accessibility, maintainability, and, more generally, on the safety and operation of a system. FMEA consists of three main components: What can go wrong? What is the probability of this failure and its effects and consequences? Can this failure be identified and detected before it occurs, and what is the probability that this will happen?

This technique can be used to identify individual critical defects early in the design process, evaluate their reliability and applicability, and enable the system to make the necessary reforms. This is one of the main advantages of using this safety risk assessment technique [19,38].

### 2.2 Study steps

**Fig 1:**
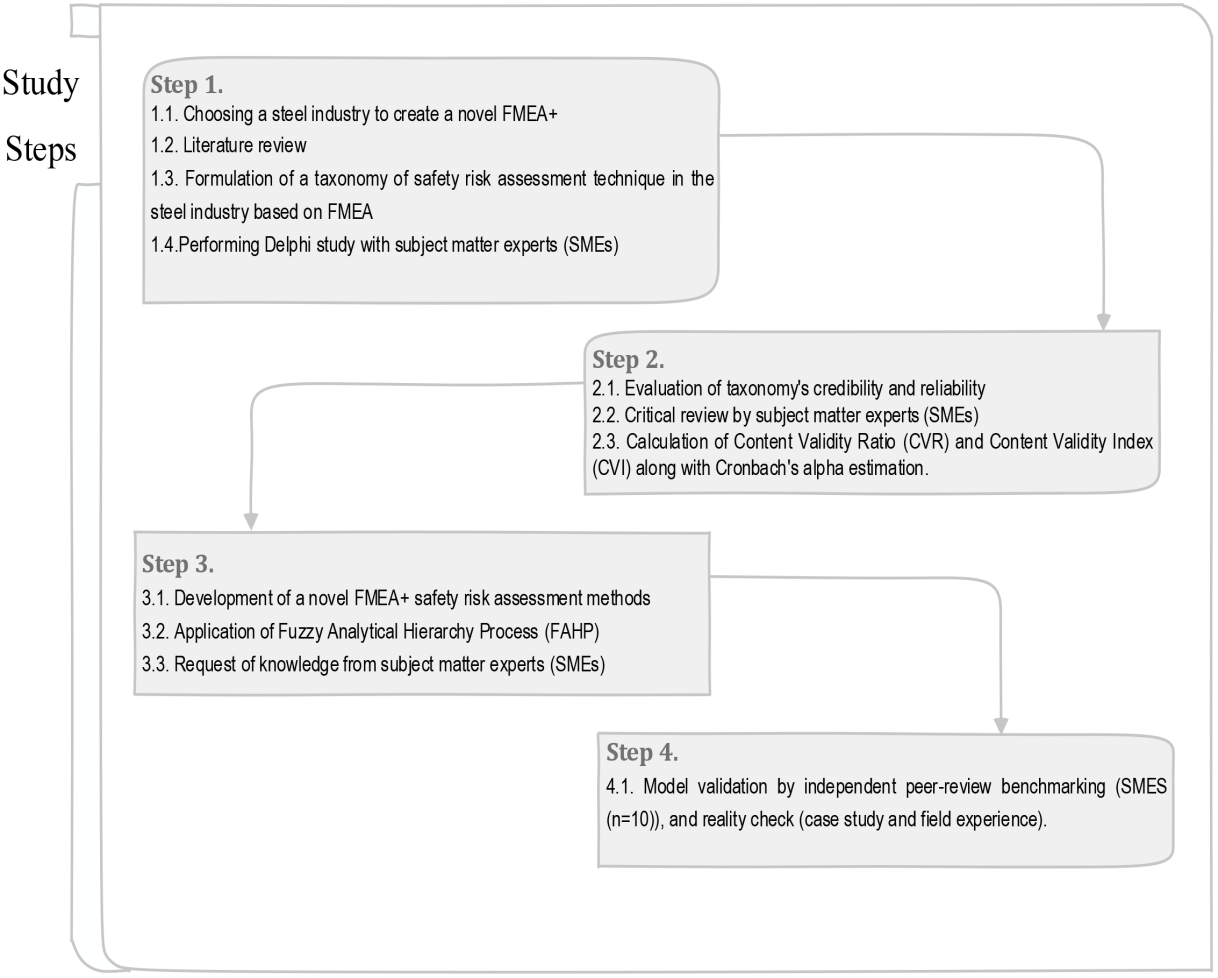
The study steps.

The conduct of the study consisted of four steps (Fig 1).

#### 2.2.1 Step 1.

This step of the study included the following phases:

- Literature review
- Formulation of a taxonomy of safety risk assessment technique in the steel industry based on FMEA
- Performing Delphi study with subject matter experts (SMEs) (N = 35)

In the first step, various FMEA studies in the steel industry and those assessing safety risk using this method were reviewed and evaluated. Then, a new taxonomy for safety risk assessment based on the FMEA technique was designed and developed as FMEA + , after reviewing the studied papers and considering the role of different parameters in the three main factors of the technique, which include probability, detectability and severity.

**2.2.1.1 Delphi method:** Delphi was developed in the 1950s and has been utilized in numerous studies. A final judgment is made by synthesizing the opinions of experts within a specific scientific domain. The Delphi method is a structured approach for forecasting future events based on expert insights across various scientific fields. Unlike traditional survey research methods, the validity of the Delphi method is not depending on the number of participants but rather on the scientific quality of those involved in the study. Typically, Delphi research includes between 5–20 participants [39]. There is also no consensus on how many rounds of Delphi should be conducted. Most studies recommend two to three rounds [40].

#### 2.2.2 Step 2.

This step of the study included the following phases:

- Evaluation of taxonomy’s credibility and reliability
- Critical review by subject matter experts (SMEs)
- Calculation of Content Validity Ratio (CVR) and Content Validity Index (CVI), along with Cronbach’s alpha estimation.

Four methods for model validation include a partial benchmark exercise, a complete benchmark exercise, independent peer review, and a reality check is recommended. To confirm the validity of the model developed in the present study, a combination of reality checks and independent peer review benchmarking was employed. Finally, the findings were examined with an expert panel and data from the studied field, along with the knowledge of subject matter experts (SMEs). In this step, three validity and reliability coefficients include Content Validity Ratio (CVR), Content Validity Index (CVI), and Cronbach’s alpha were also calculated.

#### 2.2.3 Step 3.

This step of the study included the following phases:

- Development of a novel FMEA+ safety risk assessment methods
- Application of Fuzzy Analytical Hierarchy Process (FAHP)
- Request of knowledge from subject matter experts (SMEs)

In this step, using the Analytical Hierarchy Process (AHP) and fuzzy logic, it was determined how to weight and analyze probability, detectability, severity and related parameters, and the general framework of the model was created.

**2.2.3.1 Fuzzy Analytic Hierarchy Process (FAHP):** During the present study, the Fuzzy Analytic Hierarchy Process (FAHP) was selected for its effective management of the uncertainty and imprecision inherent in expert judgments. Compared to other Multi-Criteria Decision-Making (MCDM) techniques, such as Fuzzy TOPSIS, and Fuzzy VIKOR FAHP provides superior consistency checks through pairwise comparison matrices, thereby enhancing the reliability of weight assignments. Furthermore, FAHP’s structured hierarchical framework aligns well with the multi-level risk factors considered in our study.

FAHP is particularly advantageous in decision-making situations where the consistency of pairwise comparisons is essential for achieving reliable outcomes. Unlike Fuzzy TOPSIS, Fuzzy VIKOR, and the Best-Worst Method, which focus primarily on ranking and prioritization, FAHP integrates a systematic consistency check mechanism through the calculation of consistency ratios, which help identify and rectify inconsistencies in the decision-maker’s judgments. This capability of FAHP provides a more robust and reliable framework for decision-making, ensuring that the judgment process is both accurate and consistent [3,41–43].

In addition, in the development of semi-quantitative methods for risk assessment and management, FAHP stands out as one of the easiest and most commonly used techniques. It provides an intuitive and structured approach to evaluating complex decision problems, making it a popular choice for practitioners in fields that require risk management and decision analysis. This ease of use, combined with its ability to handle uncertainty and inconsistency, further supports the suitability of FAHP in various applications [44–48].

In this study, Chang’s method was used since it provides accurate results and is simpler to implement than other methods [49]. There are different types of fuzzy numbers, and triangular fuzzy numbers are the most useful (Equation 1). The triangular fuzzy number is defined as A= (l, m, u), where l, m and u are fuzzy set identifiers. The upper bound, denoted by u, is the maximum value that fuzzy number A can take. L indicates the minimum value that fuzzy number A can assume. A fuzzy number is most likely to have m as its value [50].


μA~={*20c0,x<l(x−l)/(x−l)(m−l),\nulldelimiterspace(m−l),l≤x≤m*20c(u−x)/(u−x)(u−m),\nulldelimiterspace(u−m),m≤x≤u0x≥u (1)

It is important to note that any errors or inconsistencies in comparing and assessing the importance of options and indicators can distort the final results of the calculations. Therefore, it is crucial to verify the validity of the responses and data obtained from the experts. The experts’ answers and data were evaluated for validity based on the inconsistency rate. A consistency rate of 0.1 or less indicates consistency in the comparisons and confirms the reliability of the respondents [49]. Thus, the inconsistency rate was calculated for all pairwise comparison matrices, and invalid responses were excluded.

The information and experts’ opinions are collected and checked for validity, and a pairwise comparison matrix, which is an average of the experts’ opinions, is derived for each level. Various methods are used to calculate the average matrix and the relative weights of the criteria and sub-criteria, including row sum, column sum, arithmetic mean, and geometric mean. In this study, the geometric mean method is used. To calculate the weighting of the options, this method first calculates and normalizes the geometric mean of the rows. The next step is to rank the general criteria and sub-criteria by their relative weight and identify the most effective factors and sub-factors [49]. The hierarchical process calculates the final weighting of the options by multiplying the importance of the criteria by the weight of the options. A “priority vector” can be created by considering all judgments at all levels of the hierarchy using the “principle of hierarchical composition” [50].

In summary, the FAHP was conducted as follows:

Hierarchical Model Construction: The criteria and sub-criteria were organized hierarchically based on the results of the Delphi study. The main factors—Occurrence, Severity, and Detectability—each included four sub-factors, creating a comprehensive multi-level decision framework.

Expert Judgment Collection through Pairwise Comparisons: A panel of 40 experts conducted pairwise comparisons of criteria and sub-criteria, articulating their preferences using linguistic terms that were mapped to triangular fuzzy numbers. This methodology enabled the experts to convey uncertainty and partial confidence in their evaluations.

Consistency Assessment of Expert Inputs: Each expert’s pairwise comparison matrix was evaluated for consistency using the Consistency Ratio (CR).

The aggregation of valid judgments involved combining the consistent expert matrices using the geometric mean method. This approach produced a collective fuzzy comparison matrix for each hierarchical level, which reflects the consensus view.

Representation by Triangular Fuzzy Numbers: Linguistic scales were converted into triangular fuzzy numbers (l, m, u), which represent the lower bound, the most likely value, and the upper bound, respectively. This approach effectively captures the range of uncertainty inherent in expert judgments.

Fuzzy Synthetic Extent Calculation: Using Chang’s extent analysis method, fuzzy synthetic extents were computed for each criterion and sub-criterion. This step involves fuzzy arithmetic operations to calculate priority weights while preserving the inherent fuzziness.

Defuzzification and Normalization: The fuzzy weights were defuzzified into crisp scores using the centroid method, followed by normalization to ensure that the sum of weights at each hierarchical level equaled one.

Final Consistency Verification: The aggregated fuzzy comparison matrices exhibited low inconsistency indices (e.g., CRg = 0.025, CRm = 0.015 for the main factors), thereby confirming the reliability and stability of the aggregated judgments.

The computation of global weights involved multiplying the local weights of sub-factors by their corresponding parent factor weights, in accordance with the hierarchical composition principle. This process resulted in the determination of global priority weights.

Integration into FMEA+ Risk Priority Number Calculation: The derived weights for Occurrence, Severity, and Detectability were incorporated into the innovative FMEA+ risk priority number (RPN) formula.

#### 2.2.4 Step 4.

This step of the study included the following phase:

- Model validation by independent peer-review benchmarking (SMES (n = 10)), and reality check (case study and field experience).

Ultimately, in the fourth step, to confirm the validity of FMEA + , a mix of independent peer-review benchmarking (SMEs), and reality check (field study) were employed. Finally, the findings were investigated with the expert’s panel and data of the studied field and SMEs’ knowledge.

## 3. Results

This study was conducted with the active participation and contributions of 35 experts in various fields: occupational health and safety engineering (n = 13), mechanical and repair engineering (n = 9), production management and supervision (n = 6), and process engineering (n = 7). The average age of the experts was 41.72 ± 4.87 years, and their average work experience was 14.50 ± 4.26 years. Among the participants, 22.86% held a doctorate degree (n = 8), 37.14% had a master’s degree (n = 13), and 40.0% possessed a bachelor’s degree (n = 14).

### 3.1 Proposing a taxonomy of FMEA+ safety risk assessment method

#### 3.1.1 Delphi study.

There were three rounds in this Delphi study:

#### 3.1.2 The first round of Delphi.

In this round, various studies in the field of FMEA technique and studies that have applied this method to assess safety risks in the steel industry, were reviewed and evaluated. Subsequently, based on the analyzed papers and considering the significance of various parameters within the three primary components of this technique—probability, detectability, and severity—a new algorithm for safety risk assessment, termed FMEA + , was designed and developed. This algorithm incorporates three main factors and twelve sub-factors.

In each of the three components, the following parameters were studied:

- *Occurrence* factor: four sub-factors including reliability, system redundancy, learning from incidents, and human reliability.
- *Severity* factor: four sub-factors including human injury, financial loss (financial damage, legal fine), operational interruption (disruption of production and service operations), and reputational damage.
- *Detectability* factor: five sub-factors, including maintenance and repair data, technical inspection, daily and routine inspection, worker’s participation, and permit data. Definitions related to each of the factors and sub-factors examined are presented in Table 1.

**Table 1:** Definitions related to each of the factors and sub-factors in FMEA+ [1,44,60–65].

Factor	Definition
Occurrence	Occurrence refers to the likelihood of a specific risk occurring in certain period.
**Sub-factor**	
Reliability	Reliability in risk assessment is defined as the probability that a system, product, or component will perform its intended function without failure over a specified period under designated operating conditions.
Redundancy	Redundancy refers to the implementation of duplicate systems, processes, or controls designed to mitigate risks by providing backup in case of failure.
Incidents learning	Incident learning refers to the process through which organizations analyze and understand negative safety events, such as accidents or near misses, to prevent similar occurrences in the future.
Human reliability	Human reliability refers to the probability that a human operator will perform a task correctly and effectively under specified conditions. This concept encompasses the likelihood of successful human performance, considering various factors that can influence outcomes, such as individual capabilities, task complexity, and environmental conditions.
**Factor**	
Severity	Risk severity indicates the degree of negative impact that a risk event is likely to have on an organization’s objectives (The extent of losses and damages if potential hazards materialize).
**Sub-factor**	
Human injury	Human injury refers to the physical harm or damage that an individual may experience as a result of exposure to hazards in the workplace or environment. This concept encompasses various types of injuries, ranging from minor incidents requiring first aid to severe injuries that could lead to permanent disability or fatality
Financial loss	Financial loss refers to the potential monetary loss that an organization may incur as a result of risks materializing. This loss can arise from various factors, including market fluctuations, operational failures, or external economic conditions.
Operational interruption	Operational interruption refers to a disruption or halt in the normal functioning of an organization’s operations due to various risks or unforeseen events. This can result from factors such as system failures, human errors, natural disasters, or other operational risks that impede business processes.
Reputation damage	Reputation damage refers to the loss of financial, social, or market capital resulting from harm to an organization’s reputation. This damage can occur due to negative publicity, ethical breaches, or operational failures, and it often leads to decreased customer trust, reduced sales, and increased operational costs.
**Factor**	
Detectability	Detectability refers to the ability to identify or detect a failure mode before it affects the organization
**Sub-factor**	
Permanent maintenance	Permanent maintenance refers to the ongoing and systematic approach to maintaining systems or equipment to ensure their reliability and safety over time. This concept emphasizes the continuous monitoring, inspection, and repair of assets to detect potential failures before they occur, thereby minimizing risks associated with operational interruptions and enhancing overall system performance.
Technical inspection	Technical inspection refers to the systematic examination of equipment, processes, or systems to verify compliance with specified technical standards and safety regulations. This inspection can involve visual assessments or the use of specialized instruments to detect potential issues or failures.
Daily/routine inspection	Daily/routine inspection refers to systematic and regular evaluations conducted to identify hazards and ensure compliance with safety standards on an ongoing basis. These inspections are typically performed at specified intervals, such as daily or weekly, and aim to detect potential issues before they lead to accidents or operational disruptions.
Permit data	Permit data refers to the information and documentation associated with a Permit to Work (PTW) system, which authorizes and controls high-risk activities within a workplace. This data includes details about the specific tasks being performed, identified hazards, safety precautions required, and the personnel involved in carrying out the work.
Employee participation	Employee participation refers to the active involvement of employees in identifying, assessing, and mitigating risks within the workplace. This participation is crucial because employees are often the most familiar with the operational processes and potential hazards they encounter daily.

As part of this round, 40 experts were asked to evaluate the importance and usefulness of these factors and sub-factors in developing FMEA+ risk assessment techniques. Additionally, they were asked to propose another factor/sub-factor. After that, the results of the first round of the Delphi study were analyzed. According to the first round of Delphi study results, 35 members of the expert panel responded regarding the importance and desirability of the factors and sub-factors affecting the risk index in the FMEA+ method (response rate = 87.5%). In addition, a sub-factor titled Personnel Observation Data was proposed for the detectability factor.

#### 3.1.3 The second round of Delphi.

In the second Delphi round, after collecting opinions and analyzing the results of the first round, possible changes were made to the designed method based on the experts’ opinions (adding a sub-factor). Participants in this round were again asked to comment on the importance and desirability of the items presented. They were also asked again if they had a suggestion for adding a factor/sub-factor to the FMEA+ risk assessment. The second round of the Delphi study was then analyzed. The experts in this round made no suggestions for this algorithm at this stage.

#### 3.1.4 The third round of Delphi.

Having analyzed the results of the second round of this study and made any necessary changes, the plan for FMEA+ risk assessment technique was again sent to the expert panel for comments. It was found that the coefficient of variation (CV) was 0.05 in this round of Delphi, much lower than the standard value set for this study (<20%) [51]. Finally, the Delphi study was terminated at this point based on the CV index. After completion of the third round of the Delphi study, and based on the acceptability criteria considered for each of the factors and sub-factors in this questionnaire or on the 5-choice Likert scale (>4), only one sub-factor, permission to use data, was removed from the detectability factor. It should be noted that the level of agreement was 80% (based on all respondents). Based on the results of three rounds of Delphi, a risk assessment technique called FMEA+ was developed (Table 2).

**Table 2:** Results of the Delphi study.

Factor	Sub-factor	Importance					Mean
		Very Low		Low	Moderate	High	Very High
**Occurrence**	Reliability	0	0	0	4	31	4.886
	Redundancy	0	0	0	3	32	4.914
	Incidents learning	0	0	0	4	31	4.886
	Human reliability	0	0	0	5	30	4.857
**Severity**	Human injury	0	0	0	4	31	4.886
	Financial loss	0	0	0	4	31	4.886
	Operational interruption	0	0	0	5	30	4.857
	Reputation damage	0	0	0	4	31	4.886
**Detectability**	Permanent maintenance	0	0	0	4	31	4.886
	Technical inspection	0	0	0	5	30	4.857
	Daily/routine inspection	0	0	0	4	31	4.886
	Permit data	0	2	13	8	12	3.743^*^
	Employee participation	0	0	0	6	29	4.829

* Removed Parameter

### 3.2 Validity and reliability

The evaluation of the validity and reliability of the FMEA+ risk assessment technique revealed that the content validity ratio (CVR) was 0.77 (the minimum acceptable CVR for the panel of 35 experts is 0.31), the content validity index (CVI) was 0.91 (the minimum acceptable CVI is 0.79) and the Cronbach’s alpha coefficient was 0.86 (0.9 ≥ α > 0.8 indicates good internal reliability).

### 3.3 Proposing a novel FMEA+ method with weighted factor and sub-factors and risk level

#### 3.3.1 Fuzzy Analytic Hierarchy Process (FAHP).

**Fig 2:**
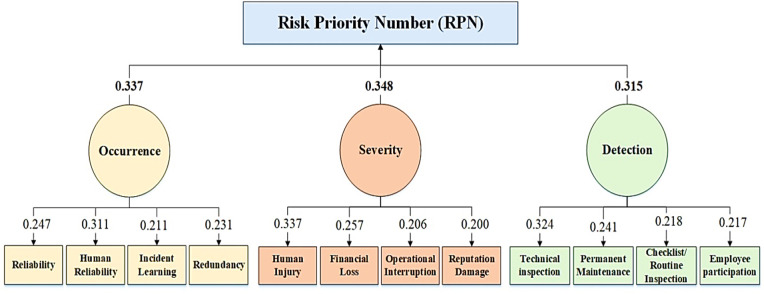
Schematic of FMEA+ method.

The weights of each factor and sub-factor were estimated using the AHP method with fuzzy logic approach following the implementation of the Delphi study and development of the FMEA+ risk assessment technique. In this step, the expert panel (40 people) received the questionnaires for the hierarchical analysis. An inconsistency rate calculation was used to evaluate the validity of each questionnaire and the pairwise comparison matrices, and invalid questionnaires were removed (5 questionnaires). Fig 2 and Tables 3–6 present the fuzzy mean matrix and the final normalized weights of the four factors of probability, severity, and detectability.

**Table 3:** Fuzzy average matrix and normalized weights of FM&EA+ technique factors.

	Severity			Detectability			Occurrence			Normalized weights
**Severity**	1.000	1.000	1.000	0.701	0.980	1.381	0.778	1.172	1.652	**0.348**
**Detectability**	0.724	1.021	1.427	1.000	1.000	1.000	0.586	0.837	1.285	**0.315**
**Occurrence**	0.605	0.853	1.295	0.778	1.195	1.705	1.000	1.000	1.000	**0.337**

**Table 4:** Fuzzy mean matrix and normalized weights of occurrence factor criteria.

	Human reliability			Reliability			Incidents learning			Redundancy			Normalized weights
**Human reliability**	1.000	1.000	1.000	0.803	1.082	1.412	1.122	1.716	2.287	0.772	1.243	1.831	**0.311**
**Reliability**	0.708	0.924	1.246	1.000	1.000	1.000	0.696	1.000	1.436	0.781	1.108	1.536	**0.247**
**Incidents learning**	0.437	0.583	0.891	0.696	1.000	1.436	1.000	1.000	1.000	0.687	0.955	1.300	**0.211**
**Redundancy**	0.546	0.804	1.296	0.651	0.903	1.281	0.769	1.047	1.455	1.000	1.000	1.000	**0.231**

**Table 5:** Fuzzy mean matrix and normalized weights of severity factor criteria.

	Human injury			Financial loss			Operational interruption			Reputation damage			Normalized weights
**Human injury**	1.000	1.000	1.000	0.910	1.268	1.601	1.195	1.785	2.287	0.835	1.324	1.904	**0.337**
**Financial loss**	0.624	0.788	1.099	1.000	1.000	1.000	0.791	1.112	1.554	0.894	1.271	1.750	**0.257**
**Operational interruption**	0.437	0.560	0.837	0.643	0.900	1.264	1.000	1.000	1.000	0.800	1.099	1.494	**0.206**
**Reputation damage**	0.525	0.755	1.197	0.571	0.787	1.119	0.669	0.910	1.250	1.000	1.000	1.000	**0.200**

**Table 6:** Fuzzy mean matrix and normalized weights of detectability factor criteria.

	Technical inspection			Employee participation			Daily/routine inspection			Permanent maintenance			Normalized weights
**Technical inspection**	1.000	1.000	1.000	0.910	1.268	1.601	1.195	1.785	2.287	0.731	1.149	1.705	**0.324**
**Employee participation**	0.624	0.788	1.099	1.000	1.000	1.000	0.665	0.924	1.326	0.686	0.942	1.326	**0.217**
**Daily/routine inspection**	0.437	0.560	0.837	0.754	1.082	1.504	1.000	1.000	1.000	0.731	1.020	1.412	**0.218**
**Permanent maintenance**	0.586	0.871	1.369	0.754	1.061	1.457	0.708	0.980	1.369	1.000	1.000	1.000	**0.241**

The fuzzy mean matrix and the final normalized weights of the three FMEA+ factors are shown in Table 3. The final normalized weights for these three factors of occurrence, severity and detectability were determined to be 0.337, 0.348, and 0.315, respectively. It should be noted that the inconsistency rate for this fuzzy matrix is CRg = 0.025 and CRm = 0.015. In other words, this matrix is highly compatible. Therefore, the calculation of RPN index in FMEA+ risk assessment technique can be calculated based on Equation 2, Fig 2 and Table 3:


RPN=(0.337 O)×(0.348 S)×(0.315 D) (2)

O: Occurrence

S: Severity

D: Detectability

The fuzzy mean matrix and the final normalized weights of the occurrence sub-factors are shown in Table 4. The final normalized weights for these four sub-factors, including reliability, system redundancy, incident learning, and human reliability, were determined to be 0.247, 0.231, 0.211, and 0.311, respectively. It should be noted that the inconsistency rate for this fuzzy matrix is CRg = 0.020 and CRm = 0.008. In other words, this matrix has a very high compatibility. Therefore, the occurrence factor can be calculated based on Equation 3 and the data presented in Table 4:


$$\text{Occurrence }=\sum {\text{O}}_{\text{i}}{\text{OW}}_{\text{i}}$$ (3)

O_i_: Estimation of Occurrence’s sub-factors

OW_i_: Weighting of Occurrence’s sub-factors

The fuzzy mean matrix and the final normalized weights of severity sub-factors are shown in Table 5. The final normalized weights for these four sub-factors, including human injury, financial damage, operational interruption, and reputational damage, were found to be 0.337, 0.337, 0.206, and 0.200, respectively. It should be mentioned that the inconsistency rate for this fuzzy matrix is CRg = 0.016 and CRm = 0.007. In other words, this matrix has a very high compatibility. The severity factor can be calculated using Equation 4 and Table 5:


$$\text{Severity }=\sum {\text{S}}_{\text{i}}{\text{SW}}_{\text{i}}$$ (4)

S_i_: Estimation of Severity’s sub-factors

SW_i_: Weighting of Severity’s sub-factors

The fuzzy mean matrix and the final normalized weights of detectability sub-factors are presented in Table 6. The final normalized weights for these four sub-factors including permanent maintenance, technical inspection, daily/routine inspection, and employee participation, were found to be 0.241, 0.324, 0.218, and 0.217, respectively. It should be mentioned that the inconsistency rate for this fuzzy matrix is CRg = 0.025 and CRm = 0.010. In other words, this matrix has a very high compatibility. Therefore, the detectability factor can be calculated using Equation 5 and Table 6:


$$\text{Detectability }=\sum {\text{D}}_{\text{i}}{\text{DW}}_{\text{i}}$$ (5)

D_i_: Estimation of Detectability’s sub-factors

DW_i_: Weighting of Detectability’s sub-factors

The risk priority number in this new technique has a range of 0.01 to 40.0. The risk priority number (RPN) thresholds presented in Table 7 were established through expert consensus during the Delphi process and are aligned with the ALARP (As Low as Reasonably Practicable) principle. These thresholds delineate three distinct risk levels: acceptable risk, Tolerable Risk (As Low As Reasonably Practicable Zone) and unacceptable risk. This classification enhances practical decision-making by categorizing risks into actionable priority levels.

**Table 7:** Safety risk decision levels in the novel FMEA+ method.

Level	Risk Level	Score
1	Acceptable risk	3.694 ≥ RPN
2	Tolerable Risk (ALARP*)	3.694 < RPN ≤ 16.550
3	Unacceptable risk	RPN > 16.550

* As Low as Reasonably Practicable.

### 3.4 The FMEA+ validation

In this phase we employed a mix of a reality check and independent peer-review benchmarking (SMEs) for method validation. Finally, the findings were examined with the expert’s panel and safety data of the field study and SMEs’ knowledge. Firstly, the findings were shared with 10 independent SMEs to examine their consistency based on their experiences and expert knowledge and experience. They approved the study results, especially three main components of FMEA+ including occurrence, severity, and detection.

Moreover, we compared the findings of FMEA+ with traditional FMEA in a case study conducted within the large steel industry. In the first step, we compared the results of the traditional FMEA and FMEA+ methods. In the second step, one year after the implementation of FMEA + , we compared the results of both methods with the actual statistics of incidents and accidents recorded in the studied steel industry. The results of the case study indicated a significant relationship between the risk levels obtained from the FMEA and FMEA+ methods (p < 0.05). Additionally, it was found that FMEA+ predicted 89% of accidents with severe damage, while traditional FMEA predicted 66% (see Table 8).

**Table 8:** Comparing the results of the FMEA and FMEA+ methods with the real accidents data in the studied industry.

Methods	Predicted risk (percentage) (All of risks)			Actual statistics of accidents (n = 71) Percentage of Prediction			*p*-value^*^
	Acceptable	Tolerable		Unacceptable	Without damage (n = 32)	Minor damage (n = 30)	Severe damage (n = 9)
FMEA	29	40	31	78	63	66	0.087
FMEA+	20	43	37	72	73	89	0.036^**^
*p*-value^*^	0.043^**^						

* Chi-square test.

** Significant relationship (*p* < 0.05).

## 4. Discussion

According to Nazaripour et al, steel industry needs to evaluate safety performance to manage the risks associated with the high volume of critical processes [52]. The objective of this study is to develop a new semi-quantitative safety risk assessment method called FMEA+ based on the extensive and comprehensive application of the FMEA method in major industries around the world.

According to the findings mentioned above, the current study comprised the following steps: First, a review and evaluation of various studies in the field of FMEA technique and studies that utilized this method to assess safety risks in the steel industry. Second, based on the review of these studies, a new algorithm for safety risk assessment, termed FMEA + , was designed and developed. This algorithm incorporated three main factors and twelve sub-factors identified during a three-stage Delphi study. Third, an AHP-Fuzzy study was designed and developed. This new algorithm, based on the FMEA technique, included the three main factors: occurrence, severity, and detectability. Finally, the validity of the model was assessed.

Based on the characteristics of different workplace situations, previous studies have shown that the application of the Delphi approach and the Fuzzy Analytic Hierarchy Process (FAHP) method can be a helpful step in the development of semi-quantitative and quantitative risk assessment methods [1]. According to a study by Soltanzadeh et al. on safety risk assessment in the construction sector, personnel in this type of work face a variety of challenges, which requires consideration of a number of factors with the correct weighting for each phase of the project [53]. During the present study, the occurrence factor includes four sub-factors: reliability, redundancy, learning from incidents, and human reliability. Human injury, financial loss (financial damage, legal fine), operational interruption (disruption in production and service operations) and reputational damage comprise the severity factor. Finally, the detectability factor includes four sub-factors: Maintenance and repair data, technical inspection, daily and routine inspection, and employee involvement (Fig 2).

An assessment of the validity and reliability of the FMEA+ risk assessment technique yielded a content validity ratio (CVR) of 0.77, a content validity index (CVI) of 0.91, and a Cronbach’s alpha coefficient of 0.86, all validated against reference values.

Table 3 shows the fuzzy mean matrix and the normalized weights of the three FMEA+ factors are presented. The final normalized weights for these three factors occurrence, severity, and detectability was 0.337, 0.348 and 0.315, respectively. It was determined that the severity and detectability components had the highest and lowest weight values, respectively. It should be noted that this fuzzy matrix has an inconsistency rate of CRg = 0.025 and CRm = 0.015. In other words, this matrix is highly compatible. In a study conducted by Kamsefidi et al. using nonlinear model, revised TOPSIS, and fuzzy logic to improve RPN calculation in FMEA, it was shown that the detectability parameter and the severity of effect parameter have weights of 0.186 (lowest weight) and 0.479 (highest weight), respectively, which is consistent with the present study [11].

Table 4 presents the fuzzy mean matrix and the normalized weights of the occurrence sub-factors. The normalized weights for reliability, redundancy, incident learning, and human reliability were 0.247, 0.231, 0.211, and 0.311, respectively. Notably, human reliability was associated with the highest weight. Ade et al. (2022) identified human reliability as a key challenge in risk assessment and the occurrence of potential hazards in the offshore industries [54]. The study conducted by Mahdinia et al. to develop a safety risk assessment method using the AHP-Fuzzy method in the construction industry also showed that human reliability is the most important factor affecting the probability component with a weight of 0.364, which is consistent with the findings of the present study [1].

The fuzzy mean matrix and the final normalized weights of severity sub-factors are presented in Table 5. The final normalized weights for these four sub-factors, including human injury, financial loss, operational interruption, and reputational damage, were found to be 0.337, 0.337, 0.206, and 0.200, respectively. It was found that the highest weight was obtained for human injury and financial loss. The study conducted by Soltanzadeh et al. showed that the most important parameters affecting the risk severity component were the human injury and cost imposition parameters with weights of 0.369 and 0.233, respectively, which is consistent with the results of the present study [53]. The fuzzy mean matrix and the final normalized weights of the detectability sub-factors are presented in Table 6. The final normalized weights for these four sub-factors including maintenance and repair data, technical inspection, daily inspection, and employee participation, were found to be 0.241, 0.324, 0.218, and 0.217, respectively. It was found that the highest weight was given to the technical inspection parameter. The use of a variety of inspection and monitoring methods at scheduled intervals has always been one of the most important methods for recording and detecting potential safety hazards in the workplace. Based on the study conducted by Ghaleh et al. in the field of risk assessment, technical inspections are one of the most important parameters of the detectability component [55]. Finally, the levels of safety risk decision-making based on RPN in FMEA+ technique have been divided into three levels.

In the field of studying FMEA method and determining its strengths and weaknesses, many studies have been conducted, including the studies by Liu et al. in 2019 [56] and Wang et al. in 2021 [57]. Traditionally, risk priority numbers (RPNs) are collected by multiplying three risk factors such as severity (S), occurrence (O), and detection (D) in the FMEA to determine the risk priority order [58]. Higher RPN values are assumed to be more significant failure modes, so more attention should be paid to them. Therefore, the normal FMEA has been heavily criticized by the authors, and among its major problems is the equal weighting of the three components of probability, severity and detection, as well as the failure to consider other important parameters appropriate to the nature of large-scale industry when calculating each of the three components and calculating the RPN from these parameters. Omidvar et al. found in their study on the risk assessment of overhead cranes that AHP-fuzzy methods can address the issues of equal weighting of risk factors, uncertainty in data (such as expert opinions), and prioritization. Compared to the traditional FMEA method, AHP-fuzzy methods eliminate failure modes and prioritize risks more effectively [59]. In addition, Zhang et al. claimed that using multiple decision-making methods using probabilistic hesitant fuzzy linguistic term sets (PHFLTSs) could be an effective way to adjust FMEA defects [16].

Finally, the evaluation of the validity of the FMEA+ results in a case study in the steel industry showed that the findings of this model had a significant relationship with the results of the traditional FMEA method as well as the actual accident statistics. We employed a mix of a reality check and independent peer-review benchmarking (SMEs) for method validation. Firstly, the findings were shared with 10 independent SMEs to examine their consistency based on their experiences and expert knowledge and experience. They approved the study results, especially three main components of FMEA+ including occurrence, severity, and detection.

Moreover, we compared the findings of FMEA+ with traditional FMEA in the case study conducted in the large steel industry. The results of the case study showed that there was a significant relationship between the percentage of risk levels obtained in the FMEA and FMEA+ methods. It was also found that 92% and 79% of accidents with severe damage were predicted by FMEA+ and FMEA, respectively (Table 8).

The FMEA+ method developed in this study considers the key risk factors that affect the scores of the three components of occurrence, severity, and detectability. The AHP-fuzzy approach makes it possible to weight these components and also the relevant parameters, reduce the effect of data uncertainty and the effect of mental errors of the expert panel according to the wide range of parameters studied, and monitor the different dimensions of the three-dimensional matrix of the FMEA+ method, and finally adjust the obtained risk levels according to the principles of risk management and the ALARP approach, which can create a novel scientific insight into the assessment of safety risks. It might also be a more suitable alternative to the traditional FMEA method in similar industries.

The present study was conducted for the first time with the aim of developing a new safety risk assessment method based on the FMEA method and taking into account the dynamic and specific conditions and characteristics of the steel industry and similar sectors. The steel industry is a highly hazardous and complex working environment, presenting a variety of potential risks that can threaten the safety of workers. In response to these challenges, various risk assessment methods have been developed over the years to identify and mitigate potential hazards. However, many of these methods often lack the necessary depth and accuracy to effectively address the unique challenges faced by the steel industry. This has created a demand for a more comprehensive approach to occupational safety risk assessment. The FMEA+ method, specifically developed for the steel industry, offers a promising solution to this issue. It combines the strengths of existing risk assessment methods with innovative techniques, making it a highly effective tool for identifying and managing occupational safety risks within this sector. Key features of the FMEA+ method include its systematic and structured approach, multi-level analysis, and the incorporation of human factors.

The application of the FMEA+ risk assessment method in the steel industry is essential for enhancing safety and operational efficiency, particularly concerning human reliability, system reliability, human and financial damage, and the technical inspection of equipment. By systematically identifying potential failure modes and their consequences, FMEA+ enables organizations to prioritize risks based on various factors and sub-factors. This is especially critical in the steel industry, where human reliability significantly influences safety outcomes; the method highlights areas where human error could lead to serious injuries or financial losses.

In the steel industry, where complex processes and heavy machinery are involved, human reliability is crucial. FMEA+ allows for the identification of potential human errors, such as incorrect operations, maintenance lapses, or insufficient training. It helps mitigate these risks through the implementation of suitable, and sufficient control measures, including enhanced training programs, clear standard operating procedures, and applying redundant systems. Furthermore, FMEA+ improves system reliability by facilitating the identification of critical equipment and processes that require regular technical inspections. Additionally, by quantifying potential human and financial damages associated with various risks, FMEA+ provides a clear framework for decision-makers to allocate resources effectively toward risk mitigation strategies.

### 4.1 Strength and limitations

**The following are some of the strengths of this study:**

Comprehensive Risk Identification: The integration of FMEA with the Delphi method and FAHP allows for a thorough identification and evaluation of potential safety risks. This approach use expert opinion to ensure that all related factors are considered.

Enhanced Decision-Making: This study used the Delphi method to obtain collective intelligence and promote consensus-building among experts, thereby improving the reliability of risk assessments and prioritization.

Quantitative Analysis: The application of FAHP provides a structured quantitative framework for weighting the importance of various risk factors and reducing uncertainty. This approach enables more informed decision-making compared to traditional qualitative methods.

Tailored to Industry Needs: By focusing on the steel industry, we created a method specifically designed to address the unique safety challenges and operational complexities inherent to this sector.

The potential for continuous improvement: The iterative nature of the Delphi method promotes the ongoing refinement of the risk assessment process, allowing organizations to adapt to new information and changing situations over time.

**The following are some limitations of this study:**

Subjectivity in Expert Opinions: The reliance on expert judgment in both the Delphi method and FAHP can introduce biases, as different experts may have varying opinion on the occurrence, severity, and detectability of risks.

Resource Intensive: Conducting a Delphi study is resource-intensive, requiring substantial time and resources to gather, and synthesize expert input. This may not be practical for all organizations.

Limited Generalizability: While the findings are specifically tailored to the steel industry, they may not be directly applicable to other industries without further adaptation. This limitation could restrict the broader applicability of the developed method.

Dependence on Data Quality: The effectiveness of risk assessment depends on the quality and availability of the data used in the analysis. Low-quality data can lead to misprioritizations.

## 5. Conclusion

In this study, we developed a new safety risk assessment algorithm, named FMEA + , building upon the Failure Modes and Effects Analysis (FMEA) technique. This innovative approach incorporates three main factors—occurrence, severity, and detectability—evaluated through a comprehensive process involving a 3-stage Delphi study and the Fuzzy Analytical Hierarchy Process (AHP-Fuzzy), resulting in 12 sub-factors. The FMEA+ method aims to contribute new scientific knowledge to the field of safety risk assessment by systematically considering the principal risk factors influencing the scoring values of occurrence, severity, and detectability. The approach involves weighting these components and other pertinent parameters using the AHP-fuzzy methodology, thereby mitigating the impact of data uncertainty and minimizing potential mental errors within the expert panel. This comprehensive investigation encompasses a broader range of parameters. The FMEA+ method further distinguishes itself by monitoring the various dimensions of its three-dimensional matrix, offering a nuanced perspective on safety risk assessment. Ultimately, the algorithm adjusts risk levels in accordance with the principles of risk management and the ALARP (As Low as Reasonably Practicable) approach. This innovative approach not only provides a novel scientific insight into safety risk assessment but also represents a more fitting alternative to the traditional FMEA method within similar industries.

## Supporting information

S1 Appendix AHP sheet for FMEA+. (DOCX)

S2 Appendix Delphi and risk assessment. (XLSX)
