# Supplementary material for: Introducing FMEA plus method for comprehensive safety risk assessment in the steel industry
Source: PLoS One. 2025 Oct 9;20(10):e0331748. doi: 10.1371/journal.pone.0331748 (PMC12510507; doi:10.1371/journal.pone.0331748)
Supplement: S1 Appendix — (DOCX) [file pone.0331748.s001.docx]

**FMEA+ Questionnaire (AHP Data Sheet)**

For risk assessment using FMEA technique, three factors of probability, severity and risk detection are used.

It seems that these three factors are different in terms of importance. Now, we request you to evaluate the importance of each factor compared to the other factor by comparing these factors two by two in the questionnaire below, according to your valuable knowledge and experience.

Keep in mind when completing the questionnaire if, for example, hypothetical information source A is more important than B and B is more important than C, then according to logic, A will definitely be more important than C. Therefore, in order to avoid contradictions in the answers, pay attention to this point when completing the questionnaires and comparing the pairs of variables.

|  | **The left criterion is more important** | | | | | | | |  | **The right criterion is more important** | | | | | | | |  |
| --- | --- | --- | --- | --- | --- | --- | --- | --- | --- | --- | --- | --- | --- | --- | --- | --- | --- | --- |
|  | Absolutely more important | in between | Much more important | in between | Relatively more important | in between | A little more important | in between | Equally important | in between | A little more important | in between | Relatively more important | in between | Much more important | in between | Absolutely more important |  |
|  | 9 | 8 | 7 | 6 | 5 | 4 | 3 | 2 | 1 | 2 | 3 | 4 | 5 | 6 | 7 | 8 | 9 |  |
| **Severity** |  |  |  |  |  |  |  | **10** | **13** | **11** |  |  |  |  |  |  |  | **Detectability** |
| **Severity** |  |  |  |  |  |  |  | **16** | **11** | **8** |  |  |  |  |  |  |  | **Occurrence** |
| **Detectability** |  |  |  |  |  |  |  | **8** | **10** | **17** |  |  |  |  |  |  |  | **Occurrence** |

**Occurrence Factor**

To obtain the occurrence factor, we may be able to use different information sources, the four important sources of this information include reliability data, redundancy data, experimental data and learning from incidents and events (Incident Learning), and human reliability are explained in table 1 and these sources are different in terms of importance. Now, we request you to evaluate the importance of each information source compared to the other source by comparing these sources two by two in the following questionnaire, according to your valuable knowledge and experience.

* Please read the explanations listed in Table 1 carefully before filling out the questionnaire below for better access to sources and making a more appropriate decision.

|  | **The left criterion is more important** | | | | | | | |  | **The right criterion is more important** | | | | | | | |  |
| --- | --- | --- | --- | --- | --- | --- | --- | --- | --- | --- | --- | --- | --- | --- | --- | --- | --- | --- |
|  | Absolutely more important | in between | Much more important | in between | Relatively more important | in between | A little more important | in between | Equally important | in between | A little more important | in between | Relatively more important | in between | Much more important | in between | Absolutely more important |  |
|  | 9 | 8 | 7 | 6 | 5 | 4 | 3 | 2 | 1 | 2 | 3 | 4 | 5 | 6 | 7 | 8 | 9 |  |
| Human Reliability |  |  |  |  |  |  |  | **11** | **17** | **7** |  |  |  |  |  |  |  | Reliability |
| Human Reliability |  |  |  |  |  |  | **1** | **9** | **15** | **8** | **2** |  |  |  |  |  |  | Incidents Learning |
| Human Reliability |  |  |  |  |  |  | **2** | **18** | **6** | **7** | **2** |  |  |  |  |  |  | Redundancy |
| Reliability |  |  |  |  |  |  | **4** | **9** | **9** | **9** | **4** |  |  |  |  |  |  | Incidents Learning |
| Reliability |  |  |  |  |  |  | **7** | **8** | **9** | **6** | **5** |  |  |  |  |  |  | Redundancy |
| Incidents Learning |  |  |  |  |  |  | **3** | **9** | **11** | **5** | **7** |  |  |  |  |  |  | Redundancy |

**Table 1: Explanation of different information sources of occurrence factor**

| **Descriptions** | **Information sources** | **Factor** |
| --- | --- | --- |
| It includes data and information related to the reliability of a device or system. The probability of an accident or injury is proportional to the value of the reliability index (P=1-R). | Reliability | **Occurrence** |
| Redundancy in engineering science is the placement of similar subsections in a system in parallel so that the overall performance of the system is guaranteed in emergency situations or in the event of a malfunction. | Redundancy |  |
| Empirical data is related to the statistics of incidents and defects that occurred previously in your work environment or other industries. In the evaluation of common qualitative and semi-quantitative risks, this index is used to determine the probability number. | Incidents learning |  |
| One of the most important causes of accidents is human error and unsafe actions directly and indirectly. Direct human errors can occur due to the inadequacy of duties, skills, awareness, experience, and knowledge, and indirect human errors can occur due to the inadequacy of management decisions and the occurrence of unsafe conditions and the effect on the reliability of manpower. | Human reliability |  |

**Severity Factor**

The severity of damage and accidents as an important factor may have different effects on different aspects of the organization. Different aspects of damage can include human damage, cost imposition (financial, legal fine), impact on time (disruption of production and service) and impact on the reputation of the organization.

According to the previous situation, in this case too, determine the importance of each dimension over the other dimension in the form of a two-by-two comparison.

* Before completing the questionnaire, read the explanations listed in Table 2 carefully.

|  | **The left criterion is more important** | | | | | | | |  | **The right criterion is more important** | | | | | | | |  |
| --- | --- | --- | --- | --- | --- | --- | --- | --- | --- | --- | --- | --- | --- | --- | --- | --- | --- | --- |
|  | Absolutely more important | in between | Much more important | in between | Relatively more important | in between | A little more important | in between | Equally important | in between | A little more important | in between | Relatively more important | in between | Much more important | in between | Absolutely more important |  |
|  | 9 | 8 | 7 | 6 | 5 | 4 | 3 | 2 | 1 | 2 | 3 | 4 | 5 | 6 | 7 | 8 | 9 |  |
| Human injury |  |  |  |  |  |  |  | **15** | **17** | **3** |  |  |  |  |  |  |  | Financial loss |
| Human injury |  |  |  |  |  |  | **3** | **9** | **15** | **8** |  |  |  |  |  |  |  | Operational interruption |
| Human injury |  |  |  |  |  |  | **2** | **18** | **8** | **7** |  |  |  |  |  |  |  | Reputation damage |
| Financial loss |  |  |  |  |  |  | **4** | **9** | **12** | **10** |  |  |  |  |  |  |  | Operational interruption |
| Financial loss |  |  |  |  |  |  | **7** | **9** | **11** | **8** |  |  |  |  |  |  |  | Reputation damage |
| Operational interruption |  |  |  |  |  |  | **3** | **9** | **14** | **9** |  |  |  |  |  |  |  | Reputation damage |

**Table 2: Explanations of different parameters under the influence of the risk of an event**

| **Descriptions** | **Information sources** | **Factor** |
| --- | --- | --- |
| It includes all kinds of physical and psychological damage to the people affected by the accident. | Human injury | **Severity** |
| Types of financial damages and calculable costs include medical costs, damage to equipment and legal fines, environmental damages, etc. | Financial loss |  |
| It includes work stoppages, project delays, as well as various disruptions that lead to lost time. | Operational interruption |  |
| The secondary effects caused by accidents include the reduction of sales and production power and share in the competitive market due to delays in production and supply and the quality of products and production services. | Reputation damage |  |

**Detectability Factor**

Hazard or defect detection capability includes a variety of mechanisms and processes that are used in an industry/organization to identify possible errors and defects that can lead to incidents. This factor includes parameters such as technical inspection data, repair data, daily inspection data and permit data.

According to the previous situation, in this case too, determine the importance of each dimension over the other dimension in the form of a two-by-two comparison.

* Before completing the questionnaire, read the explanations listed in Table 3 carefully.

|  | **The left criterion is more important** | | | | | | | |  | **The right criterion is more important** | | | | | | | |  |
| --- | --- | --- | --- | --- | --- | --- | --- | --- | --- | --- | --- | --- | --- | --- | --- | --- | --- | --- |
|  | Absolutely more important | in between | Much more important | in between | Relatively more important | in between | A little more important | in between | Equally important | in between | A little more important | in between | Relatively more important | in between | Much more important | in between | Absolutely more important |  |
|  | 9 | 8 | 7 | 6 | 5 | 4 | 3 | 2 | 1 | 2 | 3 | 4 | 5 | 6 | 7 | 8 | 9 |  |
| Technical inspection |  |  |  |  |  |  |  | **15** | **17** | **3** |  |  |  |  |  |  |  | Employee participation |
| Technical inspection |  |  |  |  |  |  |  | **9** | **15** | **11** |  |  |  |  |  |  |  | Daily/routine inspection |
| Technical inspection |  |  |  |  |  |  |  | **17** | **8** | **10** |  |  |  |  |  |  |  | Permanent maintenance |
| Employee participation |  |  |  |  |  |  |  | **9** | **13** | **13** |  |  |  |  |  |  |  | Daily/routine inspection |
| Employee participation |  |  |  |  |  |  |  | **9** | **14** | **12** |  |  |  |  |  |  |  | Permanent maintenance |
| Daily/routine inspection |  |  |  |  |  |  |  | **11** | **14** | **10** |  |  |  |  |  |  |  | Permanent maintenance |

**Table 3: Explanation of different information sources for detectability factor**

| **Descriptions** | **Information sources** | **Factor** |
| --- | --- | --- |
| Technical inspection data includes the results of various types of tests, measurements and technical inspections. Based on these data and the occurrence of defects and possible failures, a range of possible occurrences can be reached. | Permanent maintenance | **Detectability** |
| Maintenance and repair data includes defect data that is collected during the execution of maintenance programs. | Technical inspection |  |
| Daily inspection data consists of data sets collected, recorded and reported by HSE personnel or by checklists completed by operators. | Daily/routine inspection |  |
| The personnel observation report data includes a set of data such as anomaly reports, unsafe conditions, unsafe practices, and near-miss that are identified by personnel in the framework of a form/checklist and reported to the HSE unit. | Employee participation |  |
